# Mobilized Multipotent Hematopoietic Progenitors Stabilize and Expand Regulatory T Cells to Protect Against Autoimmune Encephalomyelitis

**DOI:** 10.3389/fimmu.2020.607175

**Published:** 2020-12-23

**Authors:** Sarantis Korniotis, Maud D’Aveni, Sébastien Hergalant, Hélène Letscher, Emmanuel Tejerina, Pauline Gastineau, Viviane A. Agbogan, Christophe Gras, Guillemette Fouquet, Julien Rossignol, Jean-Claude Chèvre, Nicolas Cagnard, Marie-Thérèse Rubio, Olivier Hermine, Flora Zavala

**Affiliations:** ^1^Université de Paris, Inserm U1151, CNRS UMR 8253, Institut Necker Enfants Malades (INEM), Paris, France; ^2^Université de Paris, INSERM UMR 1163, Institut Imagine, Laboratory of Cellular and Molecular Mechanisms of Hematological Disorders and Therapeutic Implications, Paris, France; ^3^Université de Lorraine, UMR 7365, IMoPA, Vandoeuvre-lès-Nancy, France; ^4^Université de Lorraine, CHRU Nancy, Hematology Department, Nancy, France; ^5^Université de Lorraine, Inserm U1256, NGERE, Vandoeuvre-lès-Nancy, France; ^6^Université de Paris, US 024 SFR Necker, Paris, France

**Keywords:** transcriptome, multiple sclerosis, IL-1β, expansion, stability, Tregs, mobilization, multipotent hematopoietic progenitors

## Abstract

Achieving immunoregulation *via in vivo* expansion of Foxp3^+^ regulatory CD4^+^ T cells (Treg) remains challenging. We have shown that mobilization confers to multipotent hematopoietic progenitors (MPPs) the capacity to enhance Treg proliferation. Transcriptomic analysis of Tregs co-cultured with MPPs revealed enhanced expression of genes stabilizing the suppressive function of Tregs as well as the activation of IL-1β–driven pathways. Adoptive transfer of only 25,000 MPPs effectively reduced the development of experimental autoimmune encephalomyelitis (EAE), a pre-clinical model for multiple sclerosis (MS). Production of the pathogenic cytokines IL-17 and GM-CSF by spinal cord-derived CD4^+^ T-cells in MPP-protected recipients was reduced while Treg expansion was enhanced. Treg depletion once protection by MPPs was established, triggered disease relapse to the same level as in EAE mice without MPP injection. The key role of IL-1β was further confirmed *in vivo* by the lack of protection against EAE in recipients of IL-1β–deficient MPPs. Mobilized MPPs may thus be worth considering for cell therapy of MS either per se or for enrichment of HSC grafts in autologous bone marrow transplantation already implemented in patients with severe refractory multiple sclerosis.

## Introduction

Autologous transplantation of hematopoietic progenitors (HSCs) is being performed in patients with severe, refractory, autoimmune diseases and singularly, multiple sclerosis (1–4). Although beneficial effects of the procedure have been ascribed to the resetting of a naïve, non-activated immune system, as well as a rebound of diverse regulatory cell subsets (5), the hypothesis that selective HSC subsets might exert an active immunoregulatory role should not be neglected.

Hematopoietic stem cells and progenitors are present in the bone marrow (BM) at various stages of differentiation. Long-term HSC (LT-HSC) and short-term HSC (ST-HSC) are endowed with self-renewing potential and upon transplantation replenish on the long-term or on the short-term, respectively, the entire blood system. They give rise to a series of multipotent hematopoietic progenitors (MPP) with decreasing self-renewal capacity, that differentiate toward committed progenitors and more mature cells of the various hematopoietic lineages. Four MPP subsets have been identified (MPP1-4) that, in addition to their c-kit and Sca-1 expression shared with LT- and ST-HSCs, can be distinguished immunophenotypically by the gain of expression of CD34, CD48, and CD135 (Flt3), the Flt3-L receptor and loss of CD150 expression (6).

Taking into consideration that HSCs are increasingly isolated from the peripheral blood after mobilization, the possibility that mobilized HSCs exhibit immunoregulatory properties was explored. Using a cocktail of hematopoietic growth factors composed of G-CSF and Flt3-ligand (Flt3-L) for a synergistic HSC mobilization from the BM to the periphery, we could show previously that MPP were enriched by mobilization and acquired the capacity to enhance the proliferation of TCR-activated Foxp3^+^ Tregs (7, 8). The promotion of Treg expansion also took place *in vivo*, and accounted for the protection against spontaneous type 1 diabetes (TID) in Non Obese Diabetic (NOD) mice induced by adoptive transfer of as few as 10,000 mobilized MPP per mouse. Both contact- and soluble factor-dependent mechanisms (7, 8) were involved in this acquired property of mobilized MPP.

Tregs play an important role in the control of tolerance in multiple sclerosis as well as in its animal model of experimental autoimmune encephalomyelitis (EAE). Considerable efforts are being produced aiming at promoting expansion of Tregs *in vitro* with IL-2, rapamycin, activation with anti-CD3/CD28 mAb-coated beads (9, 10) or with minute foreign antigen doses (11), for subsequent administration to patients with autoimmune diseases. Notably, Treg cell therapy may require billions of cells (12). Promoting the expansion of Tregs directly *in vivo* thus represents a therapeutic strategy worth of interest. Interleukin-2 (IL-2) at low dose has been demonstrated to expand preferentially Treg and numerous trials in a host of clinical settings are underway (13). However, it remains interesting to develop alternative strategies susceptible to confer highly selective expansion of Treg with no expansion of effector T-cells. We therefore investigated whether adoptive transfer of mobilized MPP could be used to protect against EAE by selectively promoting *in vivo* Treg expansion.

We herein report that MPP promote Treg proliferation and survival both *in vitro* and *in vivo*. Transcriptomic analysis demonstrated that Treg co-cultured with MPP display enhanced expression of several genes contributing to the stabilization of their regulatory function, reduced apoptosis and enhanced plasticity enabling them to effectively control neuro-inflammation. Adoptively transferred mobilized MPP effectively reduce the EAE score by an IL-1β–driven mechanism. Furthermore, the key role of Treg in the protection against EAE conferred by MPPs was demonstrated by disease relapse occurring upon depletion of Treg undertaken once protection was established. Therefore, *in vivo* expansion of functional Treg can be efficiently induced by MPP, and MPP-based cell therapy could represent a therapeutic strategy against autoimmune diseases either per se or as a an enrichment of autologous HSCT, already implemented in patients with severe multiple sclerosis (1–5).

## Materials and Methods

### Mice

Wild type C57BL/6J, C57BL/6 Foxp3-GFP-KI, and C57BL/6 IL-1β^−/−^ (obtained from CDTA, Orléans, France) mice were bred in our animal facility under specific pathogen-free conditions. Live animal experiments were conducted according to the EU Directive 2010/63/EU for animal experiments under an animal study proposal approved by the Paris Descartes University Ethical Committee for Animal Experimentation and the French Ministry of Research and Higher Education, number 3846-2015070622031545v4.

### EAE Induction

Active EAE was induced in 10- to 12-week-old female mice by s.c. immunization at two sites, upper back and lower back, with 200 μg myelin oligodendrocyte glycoprotein (MOG)_35–55_ peptide (MEVGWYRSPFSRVVHLYRNGK) emulsified in CFA containing 400 μg heat-killed Mycobacterium tuberculosis H37Ra (Hooke Laboratories, Lawrence, MA, USA), on day 0. Additionally, mice received 300 ng pertussis toxin (Hooke Laboratories, Lawrence, MA, USA) i.p. in 0.1 ml/mouse on days 0 and 1. Clinical signs of EAE were assessed daily with a 0- to 5-point scoring system, as follows: 0, no obvious changes in motor function compared to non-immunized mice; 0.5, tip of tail is limp; 1, limp tail; 1.5, limp tail and hind leg inhibition; 2, limp tail and weakness of hind legs; 2.5, limp tail and dragging of hind legs; 3, limp tail and complete paralysis of hind legs or paralysis of one front and one hind leg; 3.5, limp tail and complete paralysis of hind legs, in addition to: mouse is moving around the cage, but when placed to its side, is unable to right itself; 4, limp tail complete hind leg and partial front leg paralysis; 4.5, complete hind leg and partial front leg paralysis, no movement around the cage, mouse is not alert; 5, mouse does not move any more in the cage. Mice with score ≥4 for two consecutive days and mice with score 5 were euthanized. Disease scores over the course of the 35-d experiments were totalized for each animal, and the mean for the experimental group was expressed as a cumulative EAE score.

### *In Vivo* Mobilization Treatments and Isolation of Mobilized Multipotent Progenitors

Mobilized progenitor cells (MPP) were prepared as follows: Wild type or IL-1β^−/−^ C57BL/6J mice (8- to 12-week-old) were injected s.c. for four consecutive days with human recombinant G-CSF (200 μg/kg/day) (Zarzio 48 MU/0.5 ml, Sandoz) and recombinant murine Flt3L (20 μg/kg/day) (Immunotools, Friesoythe, Germany). Total splenocytes were magnetically sorted for c-kit^+^ cells with an automated magnetic cell sorter (Robosep, StemCell Technologies, Vancouver, BC, Canada), further stained with the mAbs directed against CD34 (BD Biosciences, Le Pont de Claix, France), Sca-1 (anti-Ly6A/E) and CD11b (eBioscience, ThermoFisher Scientific, Illkirch, France), and electronically sorted into c-kit^high^Sca-1^high^CD34^+^CD11b^-/low^ cells with the FACS Aria II cell sorter (BD Biosciences). Each mouse received intravenously 25,000 cells of the above subset at the same day of immunization with MOG_35-55_/CFA. Intravenous treatment with anti-CD25 mAb (PC61) (200 μg/ml) or the control isotype antibody was performed at day 17 of the disease, once protection by MPP was established.

### Assessment of Differentiation Potential of Sorted Mobilized MPP

Electronically sorted MPP were cultured on plates at 20,000 cells/ml, over OP9 or OP9Δ4 stromal cells at a 1:5 ratio, in OPTIMEM medium (Gibco) supplemented with 10% FCS, 1% antibiotics, 0.1% β-mercaptoethanol, SCF (1 ng/ml), Flt3L (10 ng/ml) (Immunotools), and IL-7 (8 ng/ml) (Peprotech, Neuilly-sur-Seine, France). After 7 days of incubation, cells were harvested, stained with appropriately labeled mAbs against CD4 (clone RM4-5), CD8 (clone 53-6.7), CD3 (clone 145-2C11), B220 (clone RA3-6B2), Gr1 (clone RB6-8C5), CD11c (clone HL3) all from BD Biosciences, NK1.1 (clone PK136, Sony) and CD11b (clone M1-70), ckit (CD117, clone 2B-8), Sca1(anti-Ly6A/E, clone D7) and PDCA-1 (clone eBio927) from eBioscience, and analyzed by flow cytometry for lineage determination.

### Isolation of Immune Cells from the Spinal Cord

Spinal cord isolated from control and MPP-recipient mice were incubated for 30 min in digestion buffer of DNAse and Liberase (27 WU/ml) in PBS 1× at 37°C, mixing every 5 min. EDTA (500 μl, 100 mM) was added for 1 min to end the digestion. Cells were passed through a 100 μm cell strainer, using a syringe plunger (back side) to smash the tissue. A Percoll separation was performed resuspending the cells first in 3 to 5 ml of 40% Percoll and underlayed with the same volume of 70% Percoll (in PBS) followed by centrifugation for 35 min at 1300*g* (2800 rpm) without brake. Cells at the interface were collected with a Pasteur pipette and diluted 10 times with complete medium RPMI 10% fetal cell serum (FCS), centrifuged and resuspended in 2 to 3 ml of complete medium.

### Staining of Cells for Flow Cytometry Analysis

To block nonspecific Fc receptor binding, cells were preincubated for 10 min at room temperature with FcR blocker 2.4G2 mAb. Cells were then stained with appropriately labeled mAbs against CD4 (clone RM4.5), c-kit (CD117) (clone 2B-8, eBioscience), CD11b (clone M1-70, eBioscience), Sca-1 (anti-Ly6A/E) (clone D7, eBioscience), CD34 (clone RAM34, BD Biosciences) as well as CD150 (Clone TC15-12F12.2, Sony Biotechnology, Weybridge, Surrey, UK), anti-CD48 (clone HM48-1, Biolegend) and Flt3 (clone A2F10, eBioscience). Nuclear Foxp3 and Ki67 expression was measured by FACS analysis as per manufacturer’s instructions (eBioscience). Cytokines were measured at day 4 after a cell culture of lymph node cells isolated from control or MPP-recipient mice, re-stimulated with the MOG_35_–_55_ peptide and in spinal cord cells the same day. Intracytoplasmic expression of cytokines was assessed after a 5-h stimulation with PMA (10 ng/ml) plus ionomycin (500 ng/ml) in the presence of Brefeldin A (2 mg/ml) for lymph nodes, and 3 h for spinal cord, followed by fixation/permeabilization with PFA/saponin and subsequent staining with specific antibodies including APC-labeled anti-IFN-γ (clone XMG1.2), PE-labeled anti–GM-CSF (clone MP1-22E9), APC-labeled anti-IL-17 (clone eBio17B7) (all from eBioscience) or isotype controls. Membrane and intracellular antigen expression were analyzed in a FACS Canto II cytometer (BD Biosciences) using FlowJo software (Treestar).

### Proliferation Assays

Proliferation Assays. CD4^+^CD25^high^ (all Foxp3^+^) cells isolated from the secondary lymphoid organs were magnetically sorted from the spleen of Foxp3-GFP-KI C57BL/6J mice. They were loaded with 5 μM carboxyfluorescein succinimidyl ester (CFSE) (Life Technologies) and cultured (5× 10^4^ cells per well) in RPMI medium 1640 supplemented with 5% (vol/vol) FCS (Life Technologies), 1% antibiotics, and 5 × 10^−5^ M β-mercaptoethanol. Cells were plated in 96-well round-bottomed culture plates, either alone or with sorted MPP at 1:1 T:MPP cell ratios, and stimulated with 2.5 μg/ml of anti-CD3 mAb (clone 145–2C11) and 5 μg/ml of anti-CD28 mAb (clone 37.51, eBioscience) for 4 days. Inhibitors were added at 5 to 20 µg/ml: anti-CD137L (clone TKS-1, Biolegend), anti-CD80 (clone 16-10A1, Biolegend), anti-GITRL (clone 5F1, Biolegend), anti-CD86 (clone GL-1, BD Biosciences), anti-OX40 (polyclonal goat IgG, R&D Systems), anti-TGFβ (clone 2G7, grown in our laboratory), anti-IL10 (clone JES052A5, R&D).

### Microarray Experiment

To analyze the possible mechanisms involved in mouse CD4^+^CD25^high^ regulatory T-cells (Treg cells) expansion promoted by mobilized MPP (MPP), we evaluated the transcriptomes of activated Treg extracted from simple culture (Treg control group) or from co-culture with MPPs (Treg + MPP). Treg and MPP were sorted as previously described. Treg were cultivated for 3 days with anti-CD3 and anti-CD28 alone or together with freshly purified MPP. After 3 days, CD4^+^ cells (Treg) were sorted for extraction of RNA using RNeasy Micro kit (QIAGEN). Its quality was verified in an Agilent Bioanalyzer. Total RNA was amplified and converted to biotinylated cRNA according to the manufacturer’s protocol (Illumina TotalPrep RNA Amplification Kit; Ambion). Paired biological replicates (3 for each group) were hybridized to the Sentrix BeadChips Array mouse WG-6 v2 (Illumina) and gene expression analysis was performed using GeneChip Mouse Genome 430 2.0 arrays (Affymetrix), as recommended by the manufacturer.

### Transcriptomics

Fluorescence values corresponding to raw expression data for each 6 samples were extracted from CEL files using the R oligo package (https://bioconductor.org/) with the corresponding platform definitions (pd.mouse430.2). Probe annotations were added using affycoretools (Bioconductor) with the mouse4302.db database. Internal positive and negative controls, and ambiguous or unknown probes were removed, which left 39.444 probes, corresponding to 21,108 unique and well-annotated genes. Briefly, quality control steps, data normalization, unsupervised explorations and functional annotations were conducted as described previously [https://doi.org/10.1007/s12035-018-1128-3]. Statistical analyses were achieved using linear modeling with empirical Bayes, *p values* were computed by applying a moderated two-way t-test and adjusted for false discovery rate (FDR) following the Benjamini–Hochberg procedure. Hierarchical clustering heat maps were obtained on gene-median-centered data with uncentered correlation as similarity metric. Volcano plot were rendered using EnhancedVolcano (Bioconductor). Additional pathway analyses were performed with the Reactome platform (https://reactome.org) and with ReactomePA (Bioconductor) for over-representation tests in mouse. Target enrichment were obtained with EnrichR [https://doi.org/10.1093/nar/gkw377]. For all experiments, an FDR or q-value < 0.05 indicated statistical significance.

### Statistical Analysis

Statistical analysis was performed using GraphPad Prism (GraphPad Software, La Jolla, CA). Disease curves were analyzed using two-way ANOVA test, with Bonferroni post-test. Cell proportions were analyzed using one way ANOVA with Bonferroni or Tukey’s post-test. Data are shown as mean ± s.e.m. *P* < 0.05 was considered statistically significant.

## Results

### Mobilized MPP Characterization

**Figure 1:**
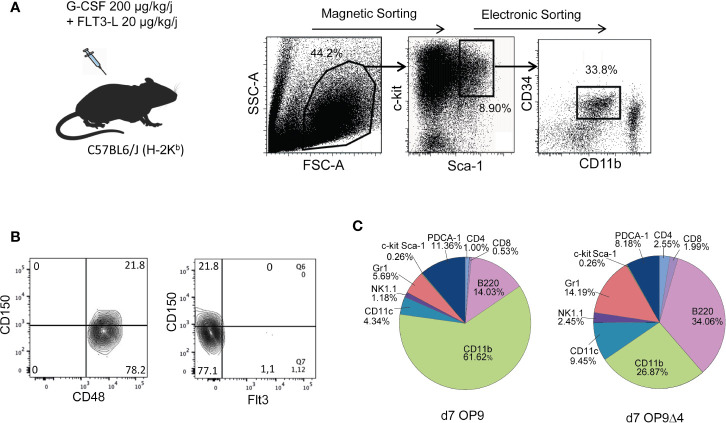
Preparation and characterization of mobilized MPP. **(A)** Upon mobilization with G-CSF and Flt3L, spleen c-kit^+^ cells were magnetically sorted, further stained with c-kit, Sca-1, CD11b and CD34 and cell-sorted as c-kit^+^Sca-1^+^ CD34^+^ CD11b^−/low^ cells. **(B)** SLAM markers including CD150 and CD48 and Flt3 were used for characterization of mobilized cell sorted c-kit^+^Sca-1^+^ CD34^+^ CD11b^−/low^ progenitors as 80% MPP3 (CD150^−^) and 20% MPP2 (CD150^+^). **(C)** The differentiation properties of mobilized MPP were assessed after 7 days of co-culture upon OP9 and OP9Δ4 stromal cells in the presence of SCF (1 ng/ml), IL-7 (8 ng/ml) and Flt3L (10 ng/ml). Cells were recovered and stained for FACS analysis with different lineage markers. Percentages of the different subsets resulting from MPP differentiation are indicated.

C-kit^+^ spleen cells derived from mobilized C57Bl/6 mice were sorted for c-kit^hi^Sca-1^hi^ CD34^+^ CD11b^−^ cells (**Figure 1A**). Using SLAM markers (14), FACS analysis revealed that 80% of the mobilized sorted cells displayed a CD48^+^CD150^−^CD135^−^(Flt3^−^) phenotype (**Figure 1B**), and thereby corresponded to the described MPP3 subset, reported to be prone to myeloid differentiation (15). Reflecting the continuum of the MPP differentiation process, approximately 20% CD150^+^ cells were detectable among the mobilized c-kit^+^Sca-1^+^CD34^+^CD11b^−^ cells, most probably corresponding to progenitors at the MPP2 (CD48^+^CD150^+^CD135^−^) stage. To assess the capacity of the sorted mobilized progenitors to differentiate into multiple hematopoietic lineages, in keeping with their MPP phenotype, we cultured them on OP-9 and OP-9Δ4 stromal cells, the latter permitting differentiation into the T-cell lineage that requires Notch pathway stimulation conferred by the expression of the Notch ligand Delta-4. A myeloid differentiation bias was also noted upon *in vitro* differentiation of the mobilized MPP. Yet, their differentiation potential into both myeloid and lymphoid hematopoietic lineages remained detectable (**Figure 1C**).

### Mobilized MPP Enhance Treg Proliferation: Transcriptomic Analysis

**Figure 2:**
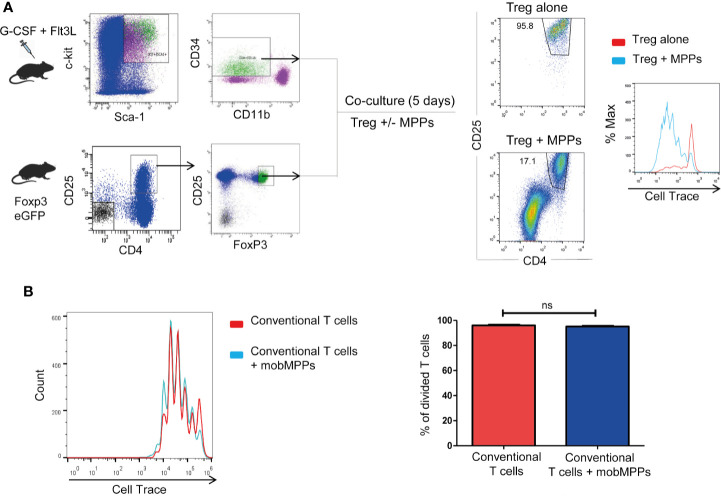
MPP promote Treg proliferation *in vitro*. Role of IL-1β. **(A)** MPP were isolated from mobilized C57BL/6 donors as in **Figure 1A** and Tregs were isolated from Foxp3 eGFP mice as CD4^+^CD25^+^ Foxp3 (GFP)^+^ cells and loaded with Cell-Trace. Tregs were stimulated for 4 days with anti-CD3/anti-CD28 in the absence or presence of MPP at a 1:1 ratio. Cell Trace incorporation was measured in the CD4^+^CD25^+^ gate. **(B)** Lack of effect of MPP co-cultured with CFSE-incorporated CD4^+^CD25^-^conventional T cells (ratio 1:1). ns: Not statistically significant by Student’s *t*-test.

**Figure 3:**
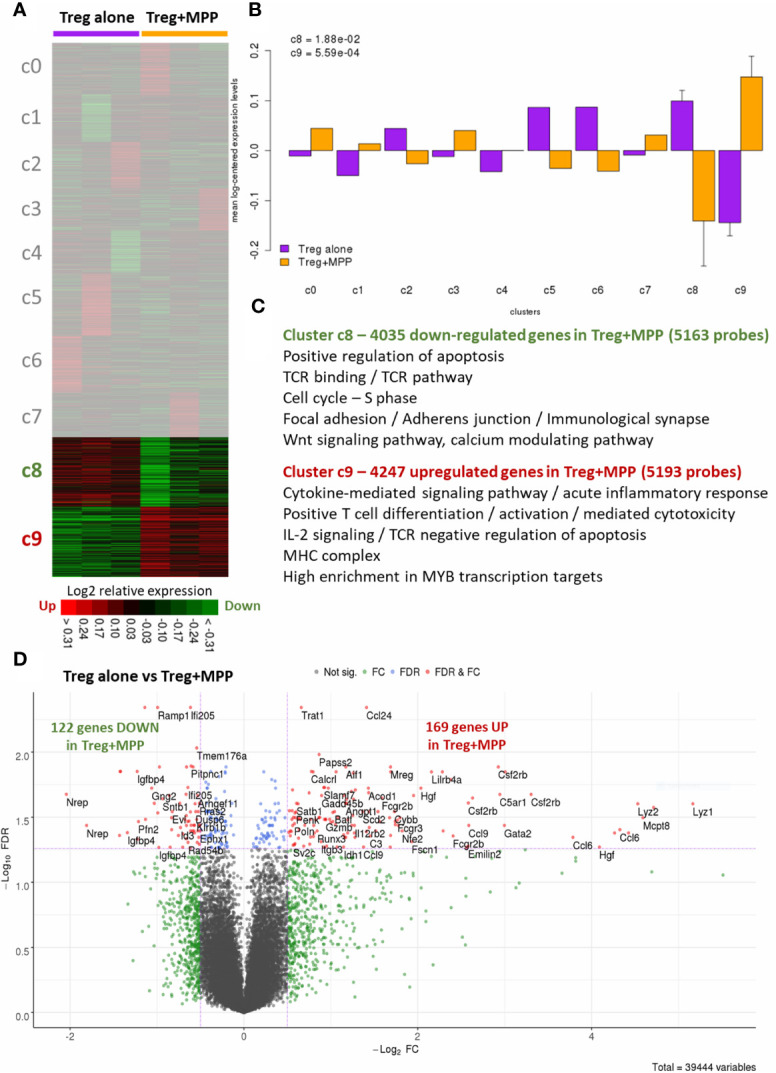
Overview of the changes induced by MPPs on the whole transcriptome (39,444 probes) of Tregs. Tregs were recovered after culture for 4 days either alone (Treg alone) or with MPPs (Treg+MPP) at a 1/1 ratio. **(A)** Unsupervised K-means clustering. The heat map delineates two large clusters of co-expressed genes (green: down-regulated genes; red: upregulated genes; black: median genes; 10 clusters labeled c0 to c9). **(B)** Grouped mean-log2-centered gene expressions in Treg alone vs. Treg+MPP for each of the 10 clusters, with standard deviation. *P* values are indicated for the significant clusters c8 and c9 (two-sided t-test). **(C)** Main enriched functional annotations for the down-regulated (c8) and upregulated (c9) K-means clusters in Treg+MPP, respectively. All FDR and q-values < 0.05. **(D)** Supervised analyses with Bayesian linear models. Volcano plot derived from the resulting statistics of Treg alone vs. Treg+MPP. FDR, False discovery rate; FC, Fold-change; FDR cutoff: 0.05; FC cutoff: −1.5 and +1.5-fold.

Treg sorted from Foxp3-GFP-KI mice and co-cultured *in vitro* at 1:1 ratio with MPP showed enhanced proliferation, assessed by CFSE dilution over 4 days in response to stimulation by anti-CD3/anti-CD28, compared to Treg cultured alone (**Figure 2A**). Furthermore, MPP did not increase the proliferation of activated CD4^+^CD25^−^ cells (**Figure 2B**). Thus, MPP specifically increased the *in vitro* expansion of TCR-activated Treg cells. Neutralization assays demonstrated that the molecular pathways (Jagged-Notch3 and GM-CSF-CD116) previously shown to be implicated in the NOD mouse (7, 8) were not implicated in the C57BL/6 strain, nor were TGF-β, IL-10, CD80, CD86, CD40, OX40, ICOS, or GITR on co-cultures, as neutralizing antibodies to these molecules had no significant effect on Treg survival and proliferation. These data raised the question about the mechanism used by MPPs to promote Treg expansion. We therefore performed microarray experiments on sorted Treg after incubation with anti-CD3/anti-CD28 either alone (Treg; N=3) or in presence of MPP during 4 days (Treg+MPP; N=3). Resulting transcriptomes finally constituted a curated dataset including 39,444 probes, corresponding to 21,108 unique genes. Unsupervised K-means clustering clearly delineated two groups of samples, with Treg on one hand and Treg+MPP on the other (**Figure 3A**). These presented with a strongly correlated gene expression profile, divided in two clusters of 4,035 and 4,247 unique genes, down- (cluster c8) and upregulated (cluster c9) in Treg+MPP, respectively.

The two dysregulated clusters represented a wide proportion (39.2%) of the whole transcriptome, with a significant differential expression between the two groups (**Figure 3B**), thus driving the sample classification. A compilation of the most relevant functional annotations, pathways and transcription factor targets (with GO, Reactome and EnrichR, respectively) were dissected for these two clusters (**Figure 3C**). Under-expressed genes in Treg+MPP (cluster c8) were mainly involved in the positive regulation of apoptosis, the Wnt calcium signaling pathway (non-canonical), or in TCR binding and signaling. The cell cycle, especially the replication during the S phase, was also found decreased, as well as the establishment of the immunological synapse through focal adhesion and adherens junction (all FDR < 0.05; **Supplementary Table S1**). This cluster presented with a large number of genes that are transcription targets of FOXO1 (n=530; q-value = 6.1e-22), an important regulator of cell death acting downstream of CDK1, PKB/AKT1, and STK4/MST1, and was also enriched in targets of FOXP3 (n = 55; q-value = 2.1e-4). Conversely, main over-expressed functions and pathways in Treg+MPP (from cluster c9) included the MHC complex, acute inflammatory response and cytokine-mediated signaling, TNFα on cytokine activity and cytokine-cytokine receptor interaction. Positive T cell differentiation, T cell activation and T cell mediated cytotoxicity were also increased in Tregs cultured with MPPs, as well as TCR negative regulation of apoptosis *via* IL-2 signaling. Glycolysis was also upregulated (**Supplementary Table S2**). Transcription targets of MYB, which prevents the differentiation of Tregs into effector T cells (16), and whose deletion leads to fatal immune pathology, were highly enriched in this cluster (n = 284; q-value = 8.7e-10).

For higher stringency and identification of core differential genes, we then performed statistical analyses with Bayesian linear models on the entire dataset and identified 291 differential genes between Treg and Treg+MPP (FDR < 0.05), 122 being down-regulated and 169 upregulated in the presence of progenitor cells, respectively (**Figure 3D**; **Supplementary Table S3**). These results completely overlapped (100%) those obtained with unsupervised methods, with wider ranges of over-expression (up to more than 30-fold) than under-expression (4-fold). Among the strongly increased genes were Lyz1 and Lyz2 (35.8-fold and 23.1-fold, respectively), coding for lysozymes related to the C-MYB transcription factor network and the innate immune system pathway. Other genes, such as Ccl6 (20-fold), coding for a small CC chemokine involved in myeloid cell recruitment, Mcpt8 (24.1-fold), predicted to be involved in granzyme-mediated apoptotic signaling pathway, and Csf2rb (9.8-fold), encoding a common subunit for the type I cytokine receptors GM-CSF, IL-3, and IL-5, were also highly increased in Tregs cultured with MPPs. On the other side, greatest decreases were observed for genes like Nrep (-4.1-fold), playing a role in the regulation of transforming growth factor beta receptor signaling pathway, Evl (-1.8-fold), a highly expressed gene in white blood cells, as well as its corresponding protein, a member of the Ena/VASP family, Igfbp4 (-2.5-fold), known to act as an apoptotic factor by reducing the growth of several cancers (17), or Bcl2l11 (-2.3-fold), coding for the BIM apoptotic activator, whose deficiency results in Treg enhanced survival and accumulation (18).

This highly specific gene signature (**Figure 3**) was explored further to study the molecular profile conferred to Treg by MPP in terms of expansion, plasticity and stabilization of their regulatory function, particularly in inflammatory settings. High expression levels were observed for Cd4, Il2ra (CD25), Il17ra (CD127), Il2rg, Ptprc (CD45), Tcrb-J, Foxp3, Foxo1, Foxo3, Lef1, Ctla4, Stat3, Cdkn1a (p21), Tgfb1, Tnfrsf1b, Tnfrsf4 (Ox40/CD134), Tnfrsf18 (Gitr), Ikzf2 (Helios), Ikzf1 (Ikaros), Nr4a3, Hif1a, and Cd28. Among these, genes with even enhanced expression after exposure to MPPs (up to 1.7-fold) were Ctla4, Tnfrsf4 (Ox40/CD134), Tnfrsf18 (Gitr), Ikzf2 (Helios), Tgfb1, Cdkn1a (p21), Stat3, Nr4a3, and Hif1a. Genes reaching high expression level status after increase included Gzmb (Granzyme B; +2-fold), Tbx21 (+2.5-fold), and Gata3 (+1.8-fold). Other increases concerned genes like Runx2, Runx3, Satb1, Cd8a (+3.9-fold), Il2, Il12rb2, Il4ra, Il10, Ifrg, Irf4, Stat5a, Prdm1, and Itgae (CD103). Cd4 expression was found to be slightly diminished in Treg+MPP but maintaining a high level, as well as Ikzf1 (Ikaros), Tcrb-J and Lef1 (down to -1.2-fold). Other highly expressed and decreasing genes included Il17ra (CD127), Il2rg, Ptprc (CD45), Foxo1, Foxo3 (-1.6-fold), Tnfrsf1b, and Cd28. Finally, Nrp1 (Neuropilin-1; -1.9-fold), Rarg, Dnmt3a, Ilr1, Wnt7a, Wnt10a, and Gata1 were also moderately down-regulated in Treg+MPP (**Supplementary Figures S1**, **S2**; **Supplementary Table S3**).

In brief, Treg exposed to MPP harbored a “specialized” molecular signature, with a stabilized phenotype Cd4^high^, Il2ra^high^, Foxp3^high^, Il7ra−, Ctla4+, Tnfrsf18+, Ikzf2+, Tnfrsf4+, Itgae+, under the transcriptional regulation of increased Satb1, Runx2, Runx3, and Gata3 gene products, switching from FOXO control, with decreased gene expression in Treg+MPP. Moreover, they exhibit enhanced expression of Prdm1 (+1.3-fold; p-value=6e-3), recently reported to prevent methylation of Foxp3 within Treg in central nervous system inflammation (19), and Nr4a (+1.45-fold; p-value = 9e-5), that stabilizes Treg against their differentiation into effector T-cells (20), which is further confirmed by the unchanged Foxp3 expression observed here after contact with MPP. Treg survival is also improved with a finely controlled and reduced apoptosis by means of TCR signaling in response to increased IL-2, the inhibition of NF-κB pathway *via* the increase of TNFα activity, the FOXO-mediated transcription, and through p21 (Cdkn1a) cell replication inhibiting activity, which has been shown to ultimately promote Treg proliferation through enhanced anti-apoptotic control (21).

**Figure 4:**
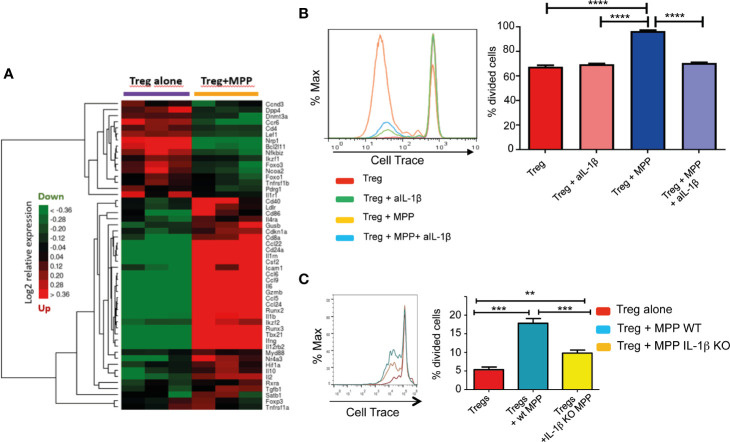
Role of the IL-1β pathway in the modulation exerted by MPPs onto Tregs. **(A)** Hierarchical clustering heat map of targeted genes in the IL-1β pathway and the inflammatory response modulated in Tregs by MPPs. The signature was obtained by compiling a list of genes included in the Il-1β/TNFα pathway from the curated gene sets on MSigDB (https://www.gsea-msigdb.org/gsea/msigdb/) and adding top correlated genes of interest to the list. Green: down-regulated genes; red: upregulated genes; black: median genes. **(B)** Role of IL-1β in enhancing proliferation of Tregs: Tregs were cultured for 4 days with anti-CD3/anti-CD28 either alone or in the presence of MPPs and a neutralizing antibody to IL-1β. Representative FACS analysis (left) and mean ± s.e.m. of triplicates (right). ****p < 0.0001 by one-way ANOVA with Bonferroni post-test. **(C)** Tregs loaded with Cell Trace were co-cultured with MPPs isolated from mobilized WT or IL-1β KO mice. Cell Trace dilution was analyzed by FACS (left) and percentages of proliferated cells compared (right: mean ± s.e.m. of triplicates from 5 cumulated experiments), **p = 0.0091, ***p < 0.001, by one-way ANOVA with Tukey’s multiple comparison test.

MPP exposed Treg also displayed molecular pathways activated by several cytokines (IL1, IL4, IL13, IL10, IL12) and several chemokines acting *via* STAT proteins (**Figure 3**; **Supplementary Tables S1**–**S3**; **Supplementary Figures S1**, **S2**). For a deeper understanding on IL-1β/TNFα−mediated inflammatory responses, we thus compiled a list of 51 genes involved and interacting with these pathways (from SigDB curated gene sets C2; https://www.gsea-msigdb.org/gsea/msigdb) along with top correlated genes extracted from gene dendrograms obtained from hierarchical clustering of each down-regulated and upregulated K-means clusters c8 and c9, respectively. Further hierarchical clustering of these 51 genes unravels two clusters of strongly correlated expression patterns, one down-regulated, and one strongly upregulated (up to 20-fold), representing an inflammatory-specific signature in Treg+MPP, completely in line with Il1b expression, but not Tnfrsf1a (**Figure 4A**). Among the genes dependent of the IL-1β pathways with enhanced expression in Treg co-cultured with MPP were Ifng (IFN-γ), Tbx21 and Ccl5 genes. In addition, pro-inflammatory Myd88 was upregulated and highly correlated with the inflammation signature.

Inflammation also affects hematopoietic progenitors. Stress hematopoiesis, triggered upon mobilization with G-CSF, prompts copious inflammatory cytokine production by MPP, including IL-1β (22), IL-1β expression is enhanced approximately 20 fold in peripheral blood CD34^+^ cells compared to BM CD34^+^ cells (23). The role of MPP-derived IL-1β in the promotion of Treg proliferation was confirmed by the reduction of the expansion effect provided by MPP onto Treg in the presence of a neutralizing antibody to IL-1β (**Figure 4B**). Moreover, efficiency of MPP isolated from IL-1β KO mice (prepared as in **Supplementary Figure S3**) in enhancing Treg proliferation *in vitro* was reduced relative to WT MPP (**Figure 4C**). Nevertheless, additional factors besides IL-1β are presumably implicated as the enhanced proliferation effect of MPP was not totally abolished in the absence or the neutralization of this cytokine.

### Adoptive Transfer of Mobilized MPP Protects Against EAE and Reduces Pathogenic Cytokine Autoimmune Production

#### Role of IL-1β

**Figure 5:**
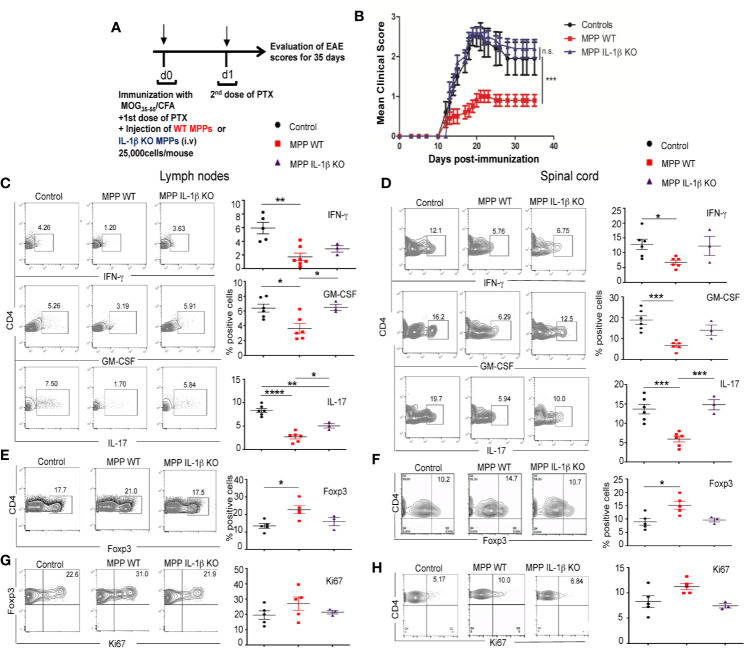
Protective role of MPPs against EAE. Role of IL-1β. **(A)** EAE was induced by s.c. injection at d0 with an emulsion of MOG_35–55_ in CFA and MPPs, isolated either from WT or IL-1β –deficient C57BL/6 mobilized donors, were adoptively transferred by retro-orbital i.v. injection (25,000 cells per recipient). A first dose of PTX was injected i.p. on d0 and a second dose on d1. **(B)** EAE clinical score was assessed daily until d35 in control mice immunized for EAE, and in mice adoptively transferred with either WT or IL-1β–deficient MPPs (n = 10 mice per group). Mean ± s.e.m., ***, *p*= 0.0015 by two-way ANOVA, with Bonferroni post-test. **(C-F)** FACS analysis of gated CD4^+^ cells isolated from cervical lymph nodes (left) and spinal cord (right) of mice from the 3 groups, at d18 after immunization. **(C, D)** Intracytoplasmic neuroinflammatory cytokine (IFN-γ, GM-CSF, IL-17) production. **(E, F)** Percentages of Foxp3 expressing CD4^+^ cells. **(G, H)** Intranuclear Ki67 expression of Foxp3^+^ CD4^+^ cells, **(C, H)** left: representative contour plots, right: summary of data. ****p < 0.0001, ***p <0.0002, **p < 0.002, *p < 0.05, by one-way ANOVA with Bonferroni post-test.

To investigate whether MPP can expand Treg *in vivo* and thereby confer protection against immune inflammatory diseases, we chose a Th1/Th17 cell-driven animal model of multiple sclerosis, experimental autoimmune encephalomyelitis (EAE). We performed the adoptive transfer of as few as 25,000 mobilized MPP per mouse, cell-sorted as shown in **Figure 1A**, at day 0 of immunization with the myelin oligodendrocyte glycoprotein peptide (MOG_35–55_) according to the administration protocol outlined in **Figure 5A**. MPP isolated from WT donors, but not from IL-1β–deficient donors (prepared as in **Supplementary Figure S3**), significantly reduced the clinical scores of EAE, relative to mice injected only with PBS (**Figure 5B**). Accordingly, both in the periphery (lymph nodes) and in the CNS (spinal cord) of MPP recipients, the CD4^+^ T-cell production of the major pathogenic cytokines, IL-17, IFN-γ and GM-CSF measured at the peak of the disease (day 21) was reduced (**Figures 5C, D**), again only in recipients of WT but not of IL-1 β–deficient MPP (**Figure 5B**). These data confirm the key role of this cytokine as well in the *in vivo* protection by MPP against the disease.

### Peripheral and CNS Treg Proliferate at Higher Rates in MPP Recipients

#### Role of IL-1β

Percentages of Foxp3^+^ Treg were enhanced in gated CD4^+^ cells in lymph nodes and spinal cord of MPP recipient mice compared to control mice (**Figures 5E, F**). Treg proliferation, measured by nuclear staining of Ki67 within gated Foxp3^+^ CD4^+^ cells at the peak of the disease tended to be enhanced in the periphery as well as in the spinal cord of MPP recipients relative to control mice with EAE, however without reaching statistical significance (**Figures 5G, H**). This *in vivo* effect on Treg was also IL-1β dependent as, contrary to recipients of WT MPP, recipients of IL-1β KO MPP showed no increase in the percentages nor in the Ki67 expression of Treg in their spinal cord, compared to non-injected EAE controls (**(Figures 5E–H**).

### The Protection Against EAE Conferred by Adoptively Transferred MPP, Once Established, Can Be Abrogated by Treg Depletion

**Figure 6:**
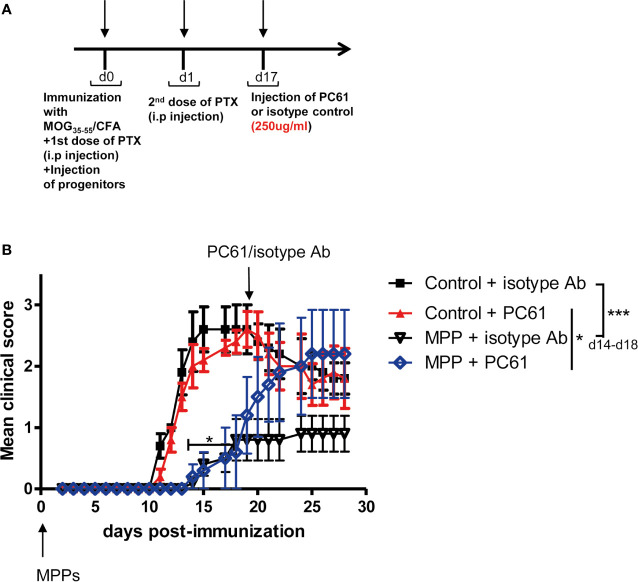
Loss of protection against EAE by MPPs upon elimination of Treg. **(A)** Mice were immunized for EAE and injected at d0 with or without MPP. At d17, control and MPP recipient mice were separated into 2 groups of 5 mice, receiving either the anti-CD25 PC61 mAb or an isotype antibody (250 μg/mouse, i.p.). **(B)** EAE was scored daily. Clinical scores are indicated as mean ± s.e.m., *p = 0.0037, ***p < 0.0001, using two-way ANOVA with Bonferroni post-test, when comparing controls with MPP recipients injected with isotype Ab. N.S. for comparisons of all other curves.

To assess whether Treg that accumulated in MPP recipients played a non-redundant role in the observed protection against disease, MPP recipient mice with established protection were divided into two separate groups (n = 5 mice per group) that received one single injection at day 17 either of the anti-CD25 PC61 mAb (250 μg/mouse, i.p.), which depletes CD25^hi^ Treg cells or of a control isotype mAb (**Figure 6A**). While the same treatments applied to control EAE undergoing mice did not change significantly the disease scores, three days after the mAb injection, disease relapse was observed in the PC61-injected group of MPP recipients which by d25 reached the clinical score of the control EAE group (**Figure 6B**). Conversely, the MPP-recipient group injected with the same amount of an isotype mAb did not significantly change its mean clinical score, and remained protected against EAE till the end of the experiment (d30). Therefore, Treg play a key role in the protection conferred by adoptively transferred MPP since their removal, after protection is established, triggers disease relapse.

## Discussion

Mobilized hematopoietic progenitor subsets at the developmental stage of MPP3 as defined by SLAM markers, that remain multipotent although with a clear myeloid developmental bias, displayed the remarkable property to promote the expansion of TCR-activated Treg, without affecting the proliferation of CD4^+^CD25^-^ cells. The functional outcomes of this property were highly significant, since as few as 25,000 mobilized MPP cells adoptively transferred per recipient conferred protection against EAE, and this protection was highly dependent on Treg as demonstrated by disease relapse upon Treg depletion by the PC61 mAb treatment in MPP-recipient mice.

The molecular mechanism by which C57Bl/6 mice derived MPPs afforded protection against autoimmune disease differs from what we had previously described in spontaneous diabetes in NOD mice in which mobilized MPP triggered both Notch- and GM-CSF-dependent signaling (7, 8). Genetic variations between strains may account for this inconsistency as it has been shown that GM-CSF effectively enhanced Treg proliferation in both the NOD and BALB/c strains but neither in the CBA nor the C57Bl/6 strains (24). Notably, both GM-CSF overproduction and persistent STAT5 phosphorylation have been reported to be amplified in autoimmune NOD bone marrow cells (25). The latter phenotype can be reproduced in non-autoimmune C57BL/6 bone marrow cultures by blocking M-CSF while stimulating with NOD level GM-CSF. Thus, the observed discrepancies between the NOD and the C57BL/6 strains in the molecular mechanisms of MPP on Treg expansion may at least in part originate in this dysregulation of GM-CSF affecting NOD myeloid cell differentiation. We herein show that in C57Bl/6 mice immunized with MOG_35–55_, IL-1β, one of the cytokines reportedly released by stress-activated MPP (22), is a key molecular mediator promoting the expansion of TCR-activated Treg both *in vitro* and *in vivo*. IL-1β likewise appears involved in the Treg expansion properties of G-CSF mobilized human MPPs isolated from healthy donors characterized in D’Aveni et al. (submitted). Yet, whether autoimmune patients will exhibit a distinct molecular mechanism for MPP expansion of Treg will have to be explored.

This may appear paradoxical since IL-1β is mostly associated with inflammation in MS and EAE. An absolute requirement of IL-1R was demonstrated for the development of EAE (26–29). IL-1β is present in MS lesions and IL-1RA moderates the induction of EAE (30, 31). Both IL-1β and IL-1RA appear in white and grey matter lesions in a model of relapsing EAE at sites of active demyelination (30). IL-1β contributes to the generation of GM-CSF^+^ Th17 cells in EAE (28, 32). An elevated IL-1β over IL-1RA ratio corresponds to higher risks of developing MS (33). In addition, IL-1β plays a role in glutamate-mediated synaptic excitability and neurotoxicity taking place in EAE (34).

Conversely, IL-1β priming reduced EAE severity in rats (28, 29, 35). Additionally, the capacity of IL-1β to enhance proliferation of TCR-activated Treg, but not of resting Treg, was recently reported in several *in vitro* studies. IL-1β has been shown to promote TGF-β and IL-2–dependent Foxp3 expression in Treg and to display co-stimulatory effects on their expansion and differentiation (36). It has been proposed that Foxp3^-^ T cells may be the primary target of IL-1β and actually support Foxp3^+^ Treg expansion (37). Here, IL-1β produced by mobilized MPP was able to enhance the proliferation and survival of Foxp3^+^ Tregs isolated from Foxp3-GFP-KI mice, thus devoid of non Treg.

Foxp3^+^ Treg are endowed with plasticity that enables them to specialize in selectively regulating effector T cell responses (38). Several of the genes dependent of the IL-1β pathways that showed enhanced expression in Treg after interaction with MPP, including Ifng (IFN-γ), Tbx21 (39), and Ccl5 (20), have been reported to confer to Treg the capacity to suppress Th1 and Th17 inflammatory cells, most appropriate to control EAE disease. Moreover, they exhibit enhanced expression of Prdm1, recently reported to prevent methylation of Foxp3 within Treg in central nervous system inflammation (19). Indeed, CD4^+^ T cells of MPP recipients displayed reduced IFN-γ, IL-17 as well as GM-CSF cytokine production. This suggests that MPP may confer to Treg the capacity to better suppress effector Tcells with the same gene set expression, i.e. Th1 and Th17 cells (38). Therefore, Treg expanded in contact with MPP have acquired the capacity to provide robust cell therapy against immune inflammatory diseases.

Moreover, they exhibit enhanced expression of Nr4a, that stabilizes Treg against their differentiation into effector Tcells (20). This stabilization is further confirmed by the high and unaffected expression level of Foxp3. Their survival is improved with a reduced apoptosis by means of TCR signaling in response to increased IL-2, the inhibition of NF-κB pathway *via* the increase of TNFα activity, the FOXO-mediated transcription, and through p21 (Cdkn1a) inhibiting activity. Their cell cycle is also slowed at the end of G1 stage through DNA synthesis inhibition in the S phase, stemming from increased p21 expression (Cdkn1a), which is in turn regulated by RUNX3 and p53. Coupled with a stringent control of apoptotic decrease, a reduced cell cycle by overexpression of Cdkn1a, and independent from Foxp3, ultimately promotes Treg generation, as was reported after ionizing radiation of Langerhans cell (21). Altogether, MPP confer to Treg stability, enhanced survival and a highly functional state along with the phenotype Cd4^high^, Il2ra^high^, Foxp3^high^, Il7ra−, Ctla4+, Tnfrsf18+, Ikzf2+, Tnfrsf4+, Itgae+.

Interaction with innate signals was reported to confer to HSC and particularly MPP the capacity to release numerous and copious amounts of cytokines and chemokines (22). Both murine and human HSC stimulated by innate signals contribute to long-term increased anti-infectious response termed trained immunity (40, 41). In systemic lupus erythematosus, an autoimmune disease with a humoral component, TLR-stimulated HSC produce IL-17 and IL-21 and expand Th17 and Tc17 cells (42), contributing to disease severity. On the other hand, hematopoietic progenitors further engaged into differentiation, in the myeloid (43), B (44–46) and pDC (Letscher et al., submitted) lineage pathways have instead been conferred immunoregulatory properties upon innate activation. Such innate activated progenitors provide protection against inflammation, autoimmune diseases, but may as well limit anti-cancer defense. They target and suppress effector T cells or favor the accumulation of diverse regulatory cell subsets. Our present data demonstrate that in organ-specific autoimmune diseases such as Type 1 diabetes and EAE, G-CSF mobilized MPP exert unique regulatory properties indirectly *via* a major mature regulatory cell type, Foxp3^+^ Treg, that they not only expand *in vivo* but also confer stabilization and fitness for controlling inflammatory settings.

The data herein presented advocate for the use of mobilized MPP as a promising tool for cell therapy of autoimmune diseases, either per se or as a complement of autologous HSC transplantation already performed in MS patients with severe, non-responsive disease. Although G-CSF was reported to provide protection in two different models of EAE in mice (47, 48), in MS patients, mobilization with G-CSF administrated alone caused disease flare (49). However, this deleterious effect could be controlled if G-CSF was associated with steroids (50). The CXCR4 antagonist plerixafor that induces rapid mobilization (51) might be worth evaluating for its potential morbidity in the MS setting and for the capacity of MPP mobilized with this alternative agent to expand Treg to the same extent as those obtained after G-CSF mobilization. In addition, the effectiveness of MPP at different disease stages remains to be evaluated. Indeed, the data presented herein demonstrate the capacity of mobilized MPP to prevent EAE induction. For a putative application to MS, their ability to reduce EAE remains to be evaluated with an adoptive transfer once clinical signs are detectable.

## Data Availability Statements

The datasets generated and analyzed for this study can be found in the GEO repository https://www.ncbi.nlm.nih.gov/geo/query/acc.cgi?acc=GSE155148. All other data are available upon reasonable request.

## Ethics Statement

The animal study was reviewed and approved by Paris Descartes University Ethical Committee for Animal Experimentation and the French Ministry of Research and Higher Education, number 3846-2015070622031545v4.

## Author Contributions

Conception: FZ. Experimental design and execution: SK, MD’A, HL, ET, PG, VA, CG, GF, and FZ. Data acquisition: SK, MD’A, HL, ET, PG, VA, CG, GF, JR, and FZ. Data analysis and interpretation: SK, MD’A, PG, ET, SH, J-CC, NC, and FZ. Preparation/revision of the manuscript: SK, MD’A, SH, MT-R, OH, FZ. All authors contributed to the article and approved the submitted version.

## Funding

FZ was supported by core funding from CNRS and INSERM and by grants received from Fondation pour la Recherche sur la Sclérose en Plaques (ARSEP) and from The Secular Society (TSS). SK and HL were supported by a Domaine d’Intérêt Majeur Biothérapies fellowship from Région Ile de France. SK was further supported by an ARSEP fellowship and Greek State Scholarship (IKY). VA was supported by a fellowship from The Secular Society (TSS).

## Conflict of Interest

The authors declare that the research was conducted in the absence of any commercial or financial relationships that could be construed as a potential conflict of interest.

## Supplementary Material

Supplementary Figure 1 Expression levels of plasticity- and stability-effector genes and their relative fold-change in Tregs cultured with MPPs when compared with Tregs alone. Mean expression levels are indicated with the standard deviation for each group (top-right corner). All FDR < 0.05; FC: Fold change; Blue: Treg alone; Orange: Treg+MPP.

Supplementary Figure 2 Expression levels of additional genes of interest in Tregs cultured with MPPs when compared with Tregs alone. Mean expression levels are indicated with the standard deviation for each group. Down-regulated and upregulated genes are all members of K-means clusters c8 and c9, respectively. All p-values < 0.05; Blue: Treg alone; Orange: Treg+MPP.

Supplementary Figure 3 Isolation of MPPs derived from IL-1b deficient C57BL/6 donor mice. Splenocytes recovered from G-CSF + Flt3L mobilized mice were magnetically sorted as c-kit+ cells, stained with CD34, Sca-1 and CD11b and sorted as c-kit+ Sca-1+ CD34+ CD11b-/low cells by flow cytometry.

Supplementary Table 1 Functional annotations of the genes in K-means cluster c8, under-expressed in Treg cultured 4 days in presence of MPP *versus* Treg alone. FDR (false discovery rate obtained by bootstrapping 50 times) cutoff is at 0.05. Enrichments in GO (Gene Ontology) terms as well as gene symbols are provided for each significant GO category. Table is sorted by decreasing enrichment.

Supplementary Table 2 Functional annotations of the genes in K-means cluster c8, over-expressed in Treg cultured 4 days in presence of MPP *versus* Treg alone. FDR (false discovery rate obtained by bootstrapping 50 times) cutoff is at 0.05. Enrichments in GO (Gene Ontology) terms as well as gene symbols are provided for each significant GO category. Table is sorted by decreasing enrichment.

Supplementary Table 3 Statistics obtained after supervised differential gene expression analyses. Moderated t-test on linear modeling with empirical Bayes was applied on Treg alone vs. Treg+MPP. FDR: False discovery rate by Benjamini-Hochberg. Fold change is indicated in plain and centered on zero.
