# Supplementary material for: Mobilized Multipotent Hematopoietic Progenitors Stabilize and Expand Regulatory T Cells to Protect Against Autoimmune Encephalomyelitis
Source: Front Immunol. 2020 Dec 23;11:607175. doi: 10.3389/fimmu.2020.607175 (PMC7786289; doi:10.3389/fimmu.2020.607175)

Supplementary Figure S1. Expression levels of plasticity- and stability-effector genes and their relative fold-change in Tregs cultured with MPPs when compared with Tregs alone. Mean expression levels are indicated with the standard deviation for each group (top-right corner). All FDR < 0.05; FC: Fold change; Blue: Treg alone; Orange: Treg+MPP.

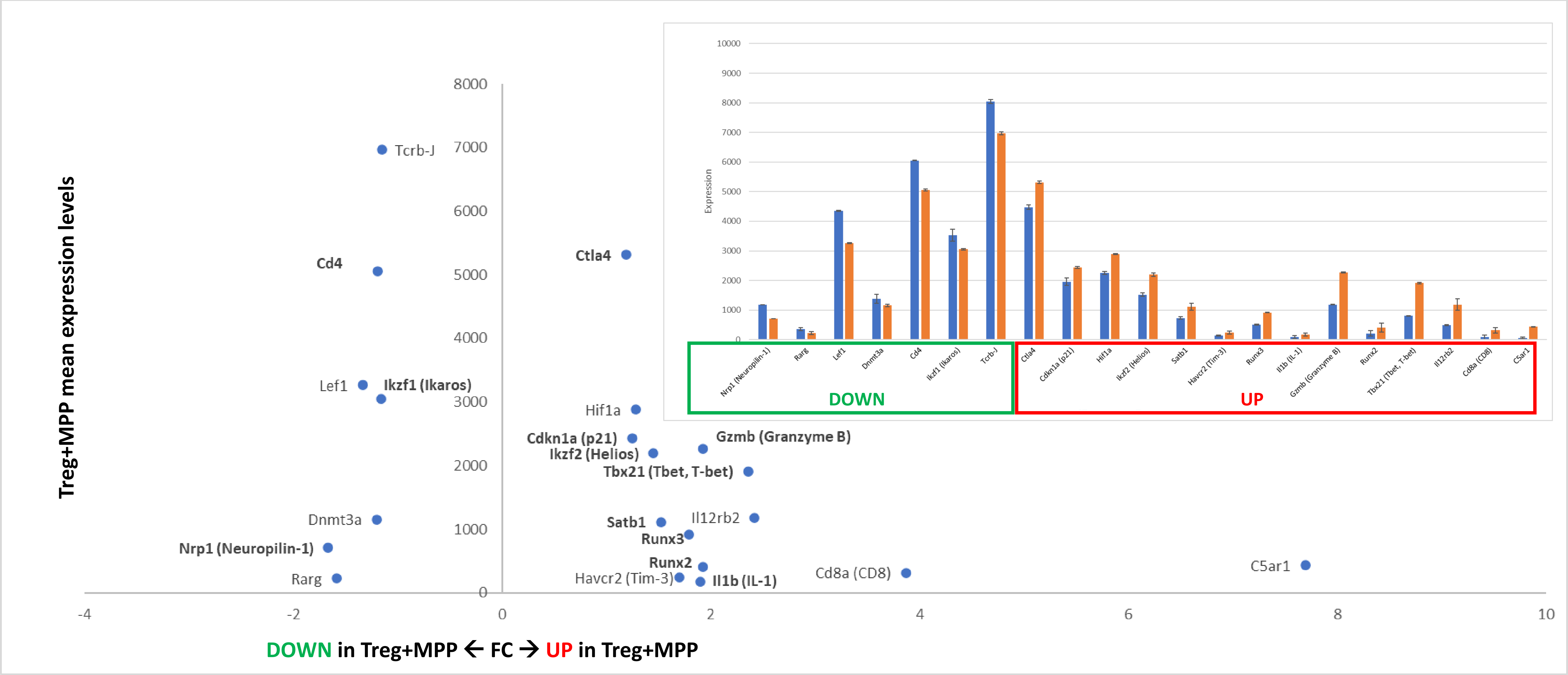

Supplementary Figure S2. Expression levels of additional genes of interest in Tregs cultured with MPPs when compared with Tregs alone.
Mean expression levels are indicated with the standard deviation for each group. Down-regulated and upregulated genes are all members of K-means clusters c8 and c9, respectively. All p-value < 0.05; Blue: Treg alone; Orange: Treg+MPP.

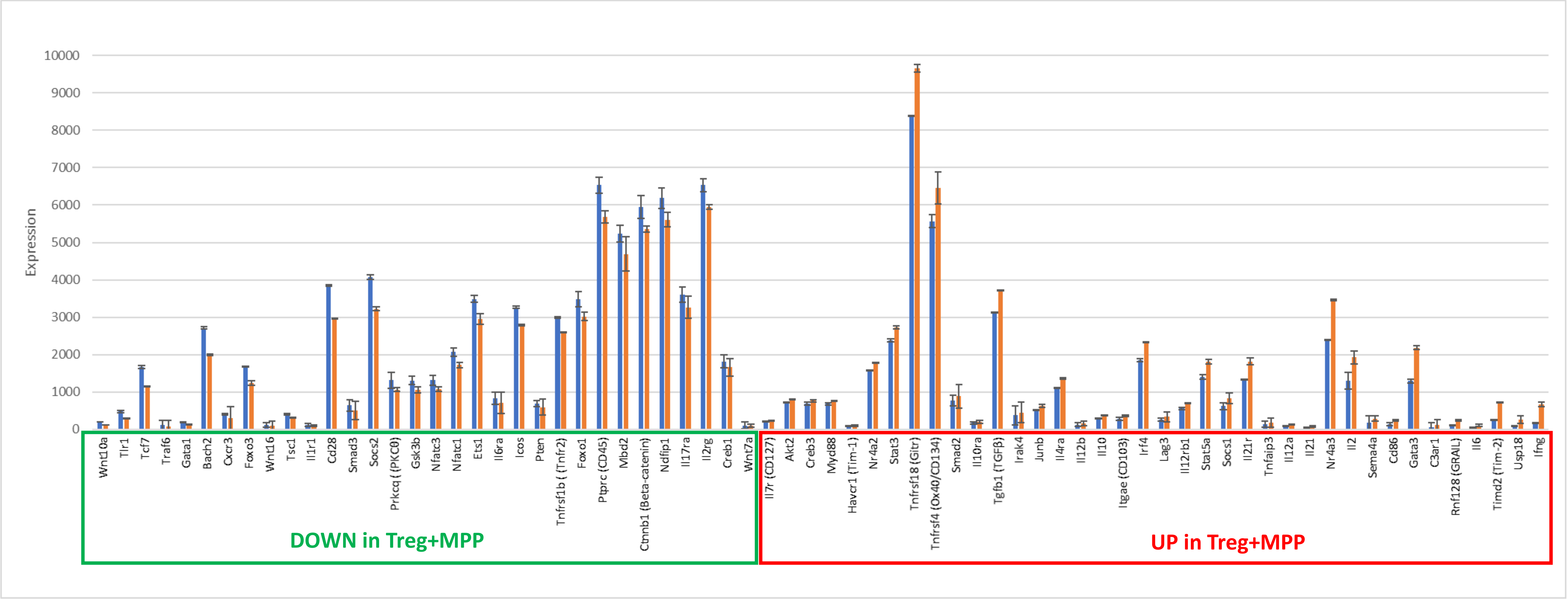

Figure S3. Isolation of MPPs derived from IL-1β deficient C57BL/6J donor mice.
Splenocytes recovered from G-CSF + Flt3L mobilized mice were magnetically sorted as c-kit$^+$ cells, stained with CD34, Sca-1 and CD11b and sorted as c-kit$^+$ Sca-1$^+$ CD34$^+$ CD11b$^{-/low}$ cells by flow cytometry.

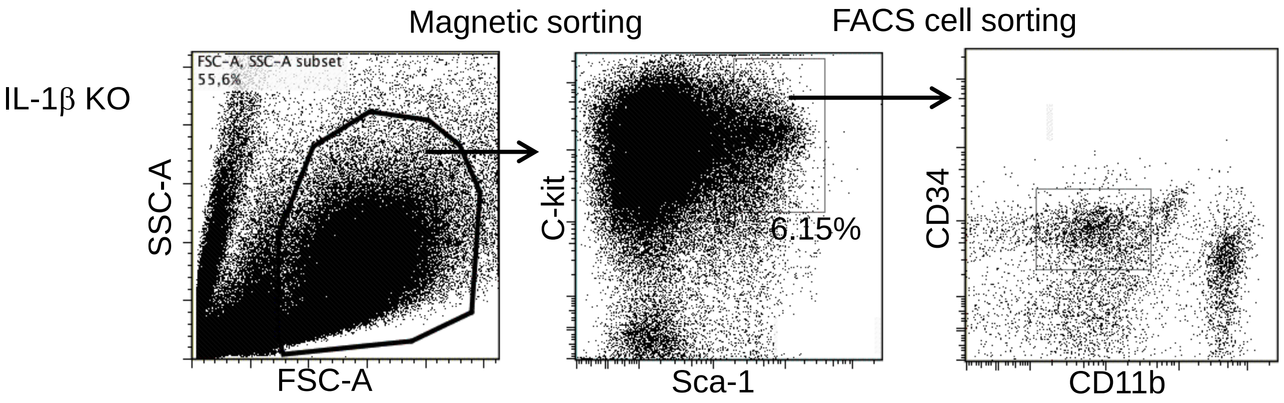

Supplement: Supplementary Figure 1 — Expression levels of plasticity- and stability-effector genes and their relative fold-change in Tregs cultured with MPPs when compared with Tregs alone. Mean expression levels are indicated with the standard deviation for each group (top-right corner). All FDR < 0.05; FC: Fold change; Blue: Treg alone; Orange: Treg+MPP. [file DataSheet_1.pdf]
